# Venous thromboembolism in acute spinal cord injury patients

**DOI:** 10.4103/0019-5413.33681

**Published:** 2007

**Authors:** Shyam K Saraf, Raj JB Rana, Om P Sharma

**Affiliations:** Department of Orthopedics, Institute of Medical Sciences, Banaras Hindu University, Varanasi, India; * Department of Radiodiagnosis, Institute of Medical Sciences, Banaras Hindu University, Varanasi, India

**Keywords:** Deep vein thrombosis, pulmonary embolism, spinal cord injury

## Abstract

**Background:**

The western literature on deep vein thrombosis (DVT) and pulmonary embolism (PE) following spinal cord injury (SCI) report an alarmingly high incidence, necessitating thromboprophylaxis. The literature on incidence from the Asian subcontinent is scanty and from India is almost nonexistent.

**Materials and Methods:**

Seventy hospitalized acute SCI patients presenting within five days of the injury were included in the present analysis. Forty-two cases were subjected to color Doppler studies and 28 cases had to be subjected to venography due to lack of facility at some point of time. The clinical course of the patients was closely observed during the period of hospitalization. All except 14 were managed nonoperatively. Thromboprophylaxis was not given to any patient at any stage; however, treatment was instituted in those showing the features of DVT on investigations.

**Results:**

Twelve patients died during the period of hospitalization. Deep vein thrombosis could be detected in seven patients only, three in the proximal and four in the distal segment of the lower limb and of these three died. Based on the clinical course and positive investigation report in favor of DVT, we presumed that the cause of death in these three patients was pulmonary embolism. In the other nine, in the absence of an autopsy report, the cause of deaths was considered as pulmonary infection, asphyxia, diaphragmatic paralysis, hematemesis, cervicomedullary paralysis etc. Clinical features to diagnose DVT were of little help.

**Conclusions:**

There is a much lower incidence (10%) of DVT and PE following spinal cord injury (SCI) in India than what is reported from the western countries. Higher age group and quadriplegia were the only factors which could be correlated. Deep vein thrombosis extending proximal to the knee was significant. In the absence of autopsy and other screening tests like D-dimer test or 125I fibrogen uptake study, the true incidence of venous thromboembolism remains uncertain. Noninvasive screening of all patients for the detection of deep vein thrombosis in SCI patients is strongly recommended.

In western countries, venous thromboembolism (VTE) is considered a major health hazard^1^–^3^ Its incidence in spinal cord injury (SCI) patients among Asians has been a topic of controversy.^4^,^5^ While some question its existence, others believe that the incidence is at par with that reported from the west.^6^,^7^ Low incidence among Asians has been attributed to several factors like high fibrinolytic activity, complete lack of Activated Protein C resistance, a higher incidence of blood group ‘O’, low intake of fat, lower incidence of obesity, climatic differences etc.^8^–^12^ However, the most important factor appears to be lack of comprehensive studies about its incidence in SCI patients in the Asian countries. Though the reports on its incidence after hip and knee surgeries have started appearing from India^13^,^14^ its incidence in SCI patients is still unreported, resulting in uncertainty about thromboprophylaxis for our patients.

## MATERIALS AND METHODS

Eighty-six patients of acute SCI were admitted between July 2002 and December 2005, of which 70 consecutive patients were considered for the study. The other 16 were excluded from the study due to associated head injury, long bone fractures, polytrauma, unconscious state. All patients with SCI presenting within five days of injury, irrespective of age and sex were included. The clinical details included nature and time of injury, period of immobilization, associated co-morbid conditions like diabetes, cardiac problem, bleeding disorders. Skeletal injury was classified region-wise and neurological status as complete (MRC Grade 0) or incomplete (MRC grade other than 0) and assessed weekly. All the patients were specifically examined for clinical evidence of DVT like fever, pedal edema, leg swelling, calf tenderness, Homan's sign, skin changes.

The Color Doppler ultrasound study was under taken in 42 patients. We used the Logic 400 (WIPRO GE) with 5 MHz linear transducer in real time B mode. Groin, thigh, popliteal fossa and leg were scanned in both longitudinal and transverse plane in both the limbs. All the three components of ultrasound examination of the vein viz. imaging, Doppler and compression were used. The results were assessed by an experienced radiologist. Our initial plans were to get the Doppler studies once during the first week and another at third week but due to various constraints, it could be done only once between 6-18 days of hospitalization. In the remaining 28 cases contrast venography was done. The absence of the normal phasic Doppler signals arising from the changes in the venous flow, incomplete color fill-in and failure to compress the vascular lumen were taken as positive signs on ultrasound color Doppler whereas contrast venography showed the filling defect due to thrombus. No fixed interval could be followed between the time of injury and investigations performed due to variable time between injury and hospitalization and appointment given by the radiologist for the said investigation.

## RESULTS

Of the 70 patients included in the study, 59 were male and 11 females. The mean age was 34 years (range16-59 years). Thirty-nine patients were of cervical spine injury resulting in quadriplegia (n=32) / quadriparesis (n=7). Nineteen of our patients developed paraplegia (MRC gr. 0) and 12 had only partial weakness of the lower limbs (MRC gr. other than 0) due to dorsal spine injury. Only two patients were found diabetic. Bedsores developed in only five patients. Of the 32 quadriplegics, five developed imaging features of DVT whereas two of the paraplegics were also diagnosed as patients with DVT. None of the quadriparetic and paraparetic patients showed any features of DVT clinically. A total of 12 patients died during the period of hospitalization. Ventilatory support could be given to only three patients, all quadriplegics. They eventually died due to severe pulmonary infection.

**Figure 1:**
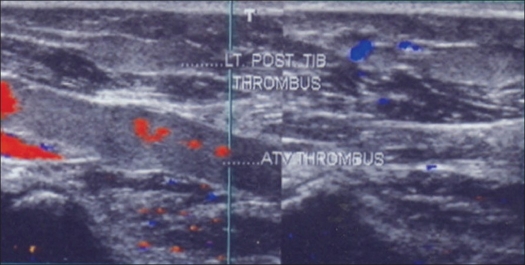
Ultrasound color Doppler showing thrombosed left anterior and posterior tibial vein

Seven of these patients were diagnosed as having DVT, four by color Doppler [Figure 1] and three by venography. Six of these patients were about 35 years age out of the 27 patients reported in this age group. Spine stabilization was done in 18 patients, of which only one developed DVT, a case of cervical C5/6 stabilization with complete quadriplegia. Among the various clinical features described, fever (n=3) and pedal edema (n=2) were the only symptoms of some significance. Six patients developed the DVT after two weeks of immobility and one in the first week itself.

Of the seven diagnosed cases of DVT, proximal DVT was diagnosed in three only. All three with proximal DVT died during the period of hospitalization. One, four and two were diagnosed DVT in the second, third and fourth week respectively. As autopsy was not allowed by relatives in any of the patients who died during the period of hospitalization, it is difficult to prove the exact cause of the death, however, the clinical features, positive findings of DVT in venography or color Doppler and sequence of symptomatology before death suggested that the cause of death could be pulmonary embolism. In the other nine deaths, the cause of death was clinically attributed to pulmonary infection, hematemesis, diaphragmatic paralysis, asphyxia, cervicomedullary paralysis etc on clinical basis. None of our patients were given thromboprophylaxis, however, the seven patients where DVT was detected were treated by injecting low molecular weight heparin (enoxaparin) in a dose of 1 mg/kg body weight twice a day subcutaneously for 10 days. Oral warfarin was started in a dose of 5 mg per day on Day 6; dose being adjusted later as per international normalization ratio (INR) value (2.0-3.0) and continued even after discharge from the hospital. Three patients died while on treatment for DVT while four were discharged.

## DISCUSSION

The incidence of DVT varying from 49-100% was documented in western literature.^1^–^3^ As the paralyzed limb lies immobile and loses the vascular tone resulting in stasis, the SCI patients are supposed to be at a higher risk of developing DVT.^15^,^16^ The incidence reported from Asian countries after various procedures in orthopedics seems to be highly variable.^17^–^19^ As controversy exists regarding its incidence, the aim of our study was to find out the incidence in our population.

We observed DVT in seven patients (10%) among SCI. The reason for this low incidence could be the genetic factor, the inherited resistance to thrombosis formation or environmental factors or maybe low socioeconomic status of our rural patients leading to a lower consumption of a fat-rich diet, the warmer climatic conditions or the practice of massage and passive exercises by attendants. The other reported factors in the literature are high fibrinolytic activity among Asians,^10^ and the prevalence of blood group ‘O’.^8^

The reported lower incidence of DVT could also be because of the lack of awareness among the doctors and the patients, and of diagnostic facilities in this part of the world; thus many of the cases remain undiagnosed. Even the investigations required to screen or diagnose such cases, like D-dimer test, Fibrinogen uptake studies, color Doppler machines or expertise to carry out venography are nonexistent in the majority of the hospitals of our country. In our study the relatively lower incidence can also be attributed to the fact that Doppler study was done only once. A more frequently carried out investigation might have given higher incidence. The timing of the investigation is also important. Rossi and colleagues^16^ documented 62% incidence on Days 6-8 and 23% between Days 9-14. It is possible to miss the diagnosis if investigations are not done at the appropriate time. It does not mean that all patients should be subjected to an exhaustive series of investigations. In fact D-dimer test or 125I fibrogen uptake study^20^ for screening and color Doppler studies or venography for further confirmations should suffice. Fujii *et al.*^4^ found no significant difference in platelet counts, mean platelet volume, fibrinogen level, von willibrand factor, platelet factor and beta thromboglobulin concentrations.

We found that the clinical features of DVT in SCI patients are of limited help for diagnosis. The features of pedal edema and associated fever are the only clinically correlating findings. Serial measurements of thigh and leg girth are not sensitive as progressive wasting of muscles or change in leg circumference may affect the result. Swarczinski *et al.*^21^ compared the daily serial calf and thigh measurements with radio fibrinogen uptake test (RFUT) and found no correlation, concluding that in SCI serial leg measurements are of no value. Akman *et al.*^22^ opined that no single clinical finding is reliably diagnostic for DVT. This situation is different following hip and knee replacement surgery, where the limb is not paralyzed. The clinical features of pulmonary embolism in quadriplegics can be misinterpreted as intercostal muscle paralysis or respiratory tract infection and thus the diagnosis of VTE can be missed. Considering the high mortality rate in our patients, particularly where the cause of death could not be attributed to other complications like renal complication, malnutrition and septicemia, chest infection, head injury, polytrauma, the diagnosis in some of the cases may be pulmonary embolism which has been missed clinically as none of our patients received thromboprophylaxis.

The literature about the incidence among quadriplegics and paraplegics is divided. Powell *et al.*^23^ did not consider any significant difference in tetraplegia and paraplegia or motor complete vs. motor incomplete lesion. Fujii^4^ (n=37) reported 38% of quadriplegics and 88% of paraplegics developing DVT. Waring and Karunas^24^ found that mortality was more among quadriplegics, complete cord lesion patients and higher age. Green^25^ concluded that high level of injury, increased BMI, lack of spasticity contribute to venous stasis and increase thromboembolic risk.

## CONCLUSION

DVT does occur in SCI in our population but there is much lower incidence than what is reported in the western literature. Higher age group and quadriplegia were the factors which could be correlated. DVT extending proximal to the knee was significant. In the absence of autopsy and other screening tests, the true incidence of VTE remains uncertain. Noninvasive screening tests like D-dimer test or 125I fibrogen uptake study of all patients for the detection of deep vein thrombosis in SCI patients is recommended.
